# Sofosbuvir-Based Therapies for Patients with Hepatitis C Virus Infection: Real-World Experience in China

**DOI:** 10.1155/2018/3908767

**Published:** 2018-11-13

**Authors:** Chengguang Hu, Guosheng Yuan, Junwei Liu, Huaping Huang, Yanyu Ren, Yinping Li, Xuefu Chen, Wei Li, Tao Wu, Hong Deng, Yanzhong Peng, Yong-Yuan Zhang, Yuanping Zhou

**Affiliations:** ^1^Department of Infectious Diseases and Hepatology Unit, Nanfang Hospital, Southern Medical University, Guangzhou, China; ^2^Department of Infectious Diseases, Peking University Shenzhen Hospital, Shenzhen, Guangdong, China; ^3^Department of Infectious Diseases, Guangdong Provincial People's Hospital, Guangzhou, China; ^4^Department of Infectious Diseases, Henan Provincial People's Hospital, Zhengzhou, Henan Province, China; ^5^Department of Infectious Disease, Hainan General Hospital, Haikou City, Hainan Province, China; ^6^Department of Infectious Diseases, The Third Affiliated Hospital of Sun Yat-Sen University, Guangzhou, China; ^7^HBVtech, Germantown, Maryland, MD 20874, USA

## Abstract

**Background and Aims:**

There is scarcity of data in literature regarding the treatment response to sofosbuvir- (SOF-) based therapies in Chinese patients with chronic Hepatitis C Virus (HCV) infection. The aim of this study was to evaluate the efficacy and safety of SOF-based regimens for chronic hepatitis C (CHC) patients without cirrhosis in a real-world setting in mainland China.

**Methods:**

A total of 226 patients receiving SOF plus daclatasvir (DCV), ledipasvir (LDV), or velpatasvir (VEL) were enrolled from December 2014 to June 2017. The primary observation point was the percentage of patients with a sustained virologic response (SVR) at posttreatment week 12 (SVR12), and all adverse events were monitored during treatment and follow-up period.

**Results:**

The overall SVR12 rate was 96% (216/226), and individual SVR12 ranged from 93% to 100% in different treatment groups. No significant differences of efficacy were detected between genotypes 1b and 6a (98% for GT 1b versus 100% for GT 6a, P=0.322). Comparing the high success rates in GT 1b and 6a patients, SVR12 was relatively low in GT 3a and 3b patients. A significant difference in efficacy was observed between GT 3 and not GT 3 patients (77% versus 98%, respectively, P<0.001). No significant differences in efficacy were detected among different regimens (93% versus 97% versus 100%, respectively, P=0.153), gender (95% for male versus 96% for female, P=0.655), or baseline HCV RNA lever (96% versus 95%, respectively, P=0.614). Similar SVR rates were also obtained in naïve and previously treated patients (98% versus 93%, respectively, P=0.100).

**Conclusions:**

NS5B polymerase inhibitor SOF plus one of the NS5A inhibitors, such as DCV, LDV, or VEL for 12 weeks was associated with high SVR12 rates and well tolerated in HCV-infected patients without cirrhosis. Moreover, patients with DAAs failure should be retreated with more effective regimens like SOF/VEL.

## 1. Introduction

Chronic HCV infection is a global health problem that affects 71 million people worldwide and 1.75 million new infections occur each year as recently estimated by World Health Organization (WHO) [1]. Pegylated-interferon (PEG-IFN) and ribavirin (RBV) combination therapy has long been the standard of care for CHC patients. However, interferon-based therapy has its own limitation because of its suboptimal sustained virologic response (SVR) rates and significant adverse events [2]. Approval and deployment of direct-acting antivirals (DAAs) in recent years have dramatically enhanced SVR response in the treated HCV chronically infected patients [3].

A standard DAA treatment consists of sofosbuvir (SOF), a HCV NS5B polymerase inhibitor (nucleotide analogue), and a HCV NS5A inhibitor, such as daclatasvir (DCV), ledipasvir (LDV), or velpatasvir (VEL). This combination offers an effective complementary mechanisms of action. The current treatment recommendations for the treatment of GT 1-6 infection from the European Association for the Study of the Liver (EASL 2016) is a combination of SOF with DCV or VEL [4]. The Asian-Pacific Association for the Study of the Liver (APASL 2016) recommends the use of SOF with LDV for treating all GTs infection but GT 3 [5].

SVR rates reported in clinical trials with the newest DAAs regimens are consistently above 95% of the treated CHC patients [6–8]. However, despite the high success rates, a small percentage of patients experience treatment failure [9, 10]. HCV resistance is largely responsible for the treatment failure and the majority of these patients harbor HCV resistant variants with resistance-associated substitutions (RASs) in the drug protein targets [9]. Recent studies suggest that preexisting RASs are associated with lower rates of virological response in certain groups of patients, such as those with GT 1 or 3 HCV, and NS5A RASs persist for a long-term after treatment failure [9, 11, 12]. These patients require other salvage regimens.

In addition, HCV genotypes show significant divergence in geographical distribution. In China, GT 1b and 2a are two major HCV subtypes, accounting for 62.78% and 17.39%, respectively. However, the major GTs in Guangdong province are 1b and 6a, accounting for 63.91% and 17.32%, respectively [13]. Although most of the DAAs-based regimens have been extensively studied, there are few published studies on Chinese population, especially lack of relevant data on GT 6a patients.

In our multicenter cohort study, we aimed to assess the efficacy and safety of 12-week therapy with SOF plus one of the NS5A inhibitors (DCV/LDV/VEL) in Chinese CHC patients without cirrhosis in a real-world setting.

## 2. Materials and Methods

**Figure 1:**
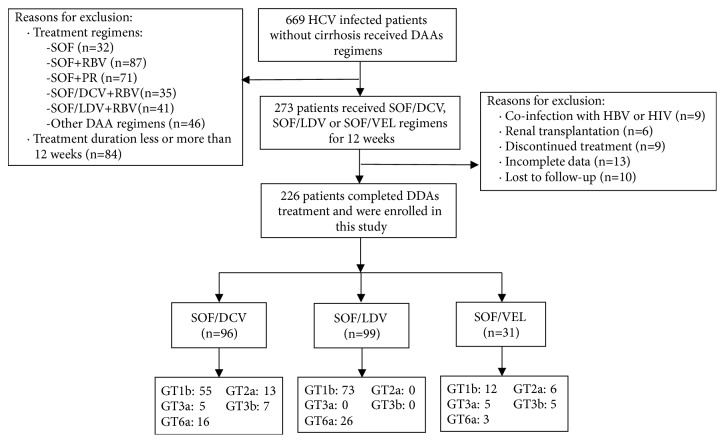
Patient recruitment flowchart.

This multicenter, retrospective real-world cohort study enrolled 669 patients with HCV infection but without cirrhosis and received DAAs therapy from December 2014 to June 2017 in (1) Nanfang Hospital, Southern Medical University, (2) Guangdong Provincial People's Hospital, (3) Peking University Shenzhen Hospital, (4) Hainan General Hospital, (5) Henan Provincial People's Hospital, and (6) The third Affiliated Hospital of Sun Yat-sen University. Of them, 226 patients with complete data met the inclusion criteria and were enrolled in final analysis (Figure 1). The study was approved by the ethical Committee of Nanfang Hospital, Southern Medical University. All procedures were carried out in accordance with the approved guidelines and the “informed” consent for the observational process was obtained from all patients prior to inclusion in the study. The observational protocol was approved by the institution's review board prior to the study initiation.

Chronic HCV-infected adults (>18 years old) without cirrhosis were eligible. Diagnosis of chronic HCV infection was based on detection of HCV RNA in serum or plasma by using the Cobras TaqMan HCV Kit (Roche Diagnostics, Indianapolis, IN, USA), with a lower limit of quantification (LLOQ) of 15 IU/ml. HCV genotyping was performed by PCR-Reverse Dot Blot. Exclusion criteria were as follows: coinfection with HBV or HIV, renal transplantation, discontinued treatment, incomplete data, or loss in follow-up during 12 weeks after treatment.

A total of 226 patients received 12 weeks of one of the SOF-based treatments: (i) SOF (400 mg once daily) + DCV (60 mg once daily); (ii) SOF (400 mg once daily) + LDV (90 mg once daily); and (iii) SOF (400 mg once daily) + VEL (100 mg once daily). Clinical, laboratory, and virological parameters were assessed at baseline and treatment weeks 4, 8, and 12 (if available), as well as 4 and 12 (if available) weeks after the end of therapy. The primary outcome was SVR12, defined as plasma HCV RNA level below quantitation limit or undetectable at least 12 weeks after treatment completion. All adverse events were monitored during the treatment and follow-up period. Virologic relapse was defined as undetectable HCV RNA at end of therapy which became detectable again thereafter without proven reinfection.

## 3. Statistical Analysis

For descriptive purposes, quantitative variables are presented as either the mean with standard deviation or medians and ranges, as appropriate. Categorical variables are presented as number and percentages. Comparisons across groups were performed using chi-squared test or Fisher's exact test for categorical variables. A two-sided* p* value of <0.05 was considered statistically significant. Statistical analysis was performed with the SPSS software, version 22.0 (IBM Corporation, Somers, NY, USA).

## 4. Results

### 4.1. Baseline Characteristics of Patients

Between December 2014 and June 2017, 669 patients were screened, of which 226 met the inclusion criteria of this study (Figure 1, appendix). Of these 226 patients, 61.9% (140/226) had GT 1b HCV infection, followed by GT 6a for 19.9% (45/226), GT 2a for 8.4% (19/226), GT 3b for 5.3% (12/226), and GT 3a for 4.4% (10/226). The mean age of the cohort was 43.4 years, 53% (120/226) were male, and 45% (101/226) were treated previously. Of the 101 previously treated patients, 73 (72%) had received treatment with a dual therapy regimen consisting of PEG-IFN and RBV, 18 (18%) had received SOF plus RBV, and 10(10%) had received a single tablet-SOF; and 52 (51%) had relapse or break through, 32 (32%) had poor or no response, and 17 (17%) were interferon intolerant. The demographic and baseline clinical characteristics of the 226 patients among three SOF-based regimens were generally balanced (Table 1).

### 4.2. Efficacy

A total of 226 CHC patients completed 12 weeks of treatment and were followed up for 12 weeks. The overall SVR12 rate was 96% (216/226), and individual SVR12 ranged between 93% and 100% in the different treatment groups. Most patients had rapid reductions in serum HCV RNA after the treatment. By the end of the fourth week of dosing, 95% (211/226) patients had HCV RNA below the limit of quantification. And by the end of treatment, HCV RNA in all of these patients remained below the limit of quantification (Table 2).

In GT 1b patients, SVR12 was 100% (55/55) for SOF/DCV group, 96% (70/73) for SOF/LDV group, and 100% (12 /12) for SOF/VEL group, while, in GT 6a patients, SVR12 was 100% among all these three groups. No significant differences in efficacy were detected between these two genotypes (98% for GT 1b versus 100% for GT 6a, P=0.322) or treatment regimens (100% versus 98% versus 100%, respectively, P=0.202). However, comparing the high success rates in GT 1b and 6a patients, SVR12 was relatively low in GT 2a, 3a, and 3b patients. Among these three groups, SVR12 was 89%, 80%, and 75%, respectively. Significant difference in efficacy was observed between GT 3 and not GT 3 patients (77% versus 98%, respectively, P<0.001). No significant differences in efficacy were detected in different treatment regimens (93% versus 97% versus 100%, respectively, P=0.153), gender (95% for male versus 96% for female, P=0.655), or baseline HCV NRA lever (96% versus 95%, respectively, P=0.614). Similar SVR rates were also obtained in naïve and previously treated patients (98% versus 93%, respectively, P=0.100) (Table 3).

Among the ten patients who did not obtain a SVR12: seven received SOF/DCV regimen (GT 2a, n=2; GT 3a, n=2; GT 3b, n=3), and the remainder received SOF/LDV regimen (GT 1b, n=3). All of these patients had undetectable HCV RNA at the end of therapy but experienced virologic relapse during the follow-up period. Most of them (7/10) were previously treated with SOF plus RBV and had poor response to them.

### 4.3. Safety

The majority of patients in each treatment group had adverse events (AEs), most of which were mild to moderate in severity. The most common AEs were fatigue (26%), headache (15%), dizziness (13%), and insomnia (11%). There were no treatment-related serious AEs and HCC during the study period (Table 4). None of the 226 patients in the study discontinued treatment owing to AEs.

## 5. Discussion

Since 2011, efficacy in the treatment of chronic hepatitis C has dramatically improved with the deployment of direct-acting antivirals (DAAs). SVR rates reported in clinical trials with different DAAs regimens are consistently above 95% of the treated HCV-infected patients [6–8]. In our study, a total of 226 patients with HCV infection received 12 weeks of treatment with SOF plus one of the NS5A inhibitors, such as DCV, LDV, or VEL. Overall SVR12 rate was 96% (216/226). Individual SVR12 ranged from 93% to 100% in the three treatment groups, which were consistent with the previously published results [6–8].

HCV GT 6 and its several subtypes are found mainly in Southeast Asia and thus far have not been well studied. The current recommendations for GT6 from the guidelines are based on small number of patients in clinical trials [14, 15]. In addition, different from other provinces, GT6a accounts for about 20.2% and 35.0% in Guangdong province and Hainan province (the two main study centers of this study), respectively [16, 17]. An important finding in this study was that the SOF-based regimens in patients infected with HCV GT 6a were as effective as those infected with GT 1b, and SVR12 was achieved by 98% and 100% in GT 1b and 6a patients (P=0.322), respectively. No significant differences in efficacy were detected either among the two genotypes with three treatment regimens (100% for SOF/DCV versus 98% for SOF/LDV versus 100% for SOF/VEL, respectively, P=0.202). Our data agreed with several studies that focused on HCV GT 6 and reported high SVR rates with SOF-based regimens: 96.0% (24/25) with SOF/LDV for 12 weeks in New Zealand [18], 100% (11/11) with SOF+RBV for 12-24 weeks in Hong Kong [19], and 95.3% (62/65) with SOF/LDV for 8-24 weeks in a Vietnamese community in USA [20]. The above data indicate that the NS5B polymerase inhibitor SOF plus one of the NS5A inhibitors, such as DCV, LDV, or VEL for 12 weeks, represents the effective treatment option for CHC patients with GT6a in Mainland China.

Another important finding in this study was that previous SOF+RBV therapy might be a risk factor for SOF+DCV treatment failure in GT3. In our entire study population, ten of 226 patients did not achieve SVR12 and half of them (5/10) were GT3 infection (GT 3a, n=2; GT 3b, n=3). SVR12 was relatively low in GT3 patients when compared with not GT 3 patients (77% versus 98%, respectively, P<0.001). Further analysis indicated that all of the 5 SOF/DCV treatment failed GT3 patients had been treated with SOF+RBV before, which was a recommended regimen under the “EASL Recommendations on Treatment of Hepatitis C 2015” but modified in the 2016 version [4, 21].

Another reason for the treatment failure in GT3 patients could be resistance-associated substitutions (RAS). HCV resistance is associated with treatment failure and most of these patients harbor HCV resistant variants with RASs in the drug protein targets [9]. Recent studies suggest that preexisting RASs result in lower rates of virological cure, and NS5A RASs persist long-term in patients after treatment failure [9, 11, 12]. Moreover, most first-generation HCV protease inhibitors and NS5A inhibitors were less effective in GT3 infection, particularly in the presence of other negative predictive factors of cirrhosis, prior interferon treatment failure, and/or RASs [11]. For example, compared to DCV, LDV was less efficient against genotypes 2–7, while VEL, a second-generation, pangenotypic HCV NS5A inhibitor, showed improved efficacy against genotypes 2 and 3 [22]. In this study, we investigated the efficacy of SOF/DCV and SOF/VEL regimen on GT 3 infection patients and we did see a significant difference in efficacy between these two regimens (58% versus 100%, respectively, P=0.02). Failure to SOF/DCV in GT3 was associated with a strong increased Y93H variant population, and it was also shown in another study, which reported that the individual RASs A30K and Y93H in GT 3 provide modest resistance to DCV and that the combinations of A30K + L31M, A30K + Y93H, A30K + L31M + Y93H provide a dramatic increase in resistance to DCV and VEL [23, 24]. Regimens like SOF/VEL demonstrated potent antiviral activity and a high barrier to resistance in vitro, and high SVR rates of 95%-99% were achieved across all HCV genotypes following 12 weeks of SOF/VEL therapy, which could be a salvage regimen for those patients with treatment failure [6, 25]. Moreover, a recent study suggested that the addition of RBV to retreatment SOF plus DCV or VEL regimens could increase SVR rates [26]. Thus, the addition of RBV may be an option for rescue treatments in difficult-to-treat GT3-infected patients as well as in DAA-naïve patients in countries in which second-generation DAA regimens are not available.

Original DAAs are unavailable in China before 2017 and only a minority of patients was able to afford the high costs. However, many patients can purchase generic or original DAAs through medical tours or other ways from neighboring countries of China. For example, in 2015, Gilead Sciences, Inc. approved generic sofosbuvir-ledipasvir with a very low price in many South Asian countries. What is more, original DAAs exported to Asian countries was much cheaper than those in Europe and America. Therefore, patients in China can go to these neighboring countries and purchase generic or original DAAs for treatment. In our study, most of patients were treated with generic DAAs produced by licensing companies, such as Indian Natco Pharma Limited and Mylan Pharma Limited. Noteworthy, many studies demonstrated that generics were well tolerated and achieved high SVR12 comparable to corresponding brand name DAAs [27–30].

The main limitations of this study were the relatively small sample size in SOF/VEL group, which resulted in a small number of patients in subgroups. In addition, we did not perform the RASs testing on those patients with DAAs failure; otherwise we may have elucidated the genetic mechanism of drug resistance. Further investigations are required to confirm our findings.

## 6. Conclusion

In conclusion, our results suggest that (1) the treatment of NS5B polymerase inhibitor SOF plus one of the NS5A inhibitors, such as DCV, LDV, or VEL for 12 weeks resulted in 96% SVR12 rates and was well tolerated in Chinese HCV-infected patients without cirrhosis and (2) patients with DAAs failure should be retreated with more effective regimens like SOF/VEL.

## Figures and Tables

**Table 1:** Baseline demographics and biochemical characteristics of study population.

	SOF+DCV N=96	SOF+LDV N=99	SOF+VEL N=31	Total N=226
Male, n(%)	43 (45%)	65 (66%)	12 (39%)	120 (53%)
Age(years), *Mean±SD*	46.6±14.3	39.6±7.8	42.7±15.2	43.4±8.3
Treatment history, n(%)				
Treatment naïve	54 (56%)	50 (51%)	21 (68%)	125 (55%)
Treatment-experienced	42 (44%)	49 (49%)	10 (32%)	101 (45%)
HCV genotype, n(%)				
1b	55 (57.2%)	73 (73.7%)	12 (38.7%)	140 (61.9%)
2a	13 (13.5%)	0	6 (19.4%)	19 (8.4%)
3a	5 (5.2%)	0	5 (16.1%)	10 (4.4%)
3b	7 (7.3%)	0	5 (16.1%)	12 (5.3%)
6a	16 (16.7%)	26 (26.3%)	3 (9.7%)	45 (19.9%)
ALT(U/L),* Mean±SD*	42.3±16.3	38.6±14.6	41.5±19.8	41.6±15.5
AST(U/L),* Mean±SD*	34.3±13.5	33.2±12.8	35.6±15.2	34.5±13.1
Hemoglobin (g/L),* Mean±SD*	136.2±20.2	128.8±31.1	133.2±22.5	133.1±30.8
Platelet×10^9^/L,* Mean±SD*	208.2±36.6	233.6±42.2	268.4±48.5	230.7±45.2
AFP (ng/ml),* Mean±SD*	6.6±3.1	5.8±3.0	5.5±2.7	6.0±3.2
HCV RNA, log10 IU/ml,* Mean±SD*	5.6±2.2	5.8±1.7	5.3±1.6	5.6±2.0
LSM (kPa),* Mean±SD*	5.9±2.3	6.0±2.1	6.4±1.8	6.0±2.3

SOF, Sofosbuvir; DCV, Daclatasvir; LDV, Ledipasvir; VEL, Velpatasvir; HCV, hepatitis C virus; ALT, alanine aminotransferase; AST, aspartate aminotransferase; AFP, alpha-fetoprotein; LSM, liver stiffness measurement.

**Table 2:** Virological response during and after treatment.

	SOF+DCV N=96	SOF+LDV N=99	SOF+VEL N=31	Total N=226
HCV RNA <15 IU/ml, n/N (%)				
During treatment				
Week 1	65/96 (68%)	71/99 (72%)	20/31 (71%)	159/226 (70%)
Week 4	88/96 (92%)	93/99 (94%)	30/31 (97%)	211/226 (95%)
Week 8	95/96 (99%)	99/99 (100%)	31/31 (100%)	225/226 (99%)
Week 12	96/96 (100%)	99/99 (100%)	31/31 (100%)	226/226 (100%)
After treatment				
SVR 4	94/96 (98%)	99/99 (100%)	31/31 (100%)	223/226 (99%)
SVR12	89/96 (93%)	96/99 (97%)	31/31 (100%)	216/226 (96%)
SVR12, n (%)				
Treatment history				
Treatment naïve	52/54 (96%)	49/50 (98%)	21/21 (100%)	122/125 (98%)
Treatment-experienced	37/42 (88%)	47/49 (96%)	10/10 (100%)	94/101 (93%)
HCV genotype				
1b	55/55 (100%)	70/73 (96%)	12/12 (100%)	137/140 (98%)
2a	11/13 (85%)	0	6/6 (100%)	17/19 (89%)
3a	3/5 (60%)	0	5/5 (100%)	8/10 (80%)
3b	4/7 (57%)	0	5/5 (100%)	9/12 (75%)
6a	16/16 (100%)	26/26 (100%)	3/3 (100%)	45/45 (100%)

**Table 3:** SVR12 by baseline factors.

	SVR12 rates		
	n/total (%)	*χ* ^2^	*P* value
Total	216/226 (96%)	—	—
By regimen		3.756	0.153
SOF+DCV	89/96 (93%)		
SOF+LDV	96/99 (97%)		
SOF+VEL	31/31 (100%)		
By gender		0.200	0.655
male	114/120 (95%)		
female	102/106 (96%)		
By treatment history		2.712	0.100
treatment naïve	122/125 (98%)		
treatment experience	94/101 (93%)		
By HCV genotype		0.908	0.322
GT 1b	137/140 (98%)		
GT 6a	45/45 (100%)		
		19.306	<0.001
GT 3	17/22 (77%)		
Not GT 3	199/204 (98%)		
By HCVRNA, log10 IU/ml		0.254	0.614
<10^6^	104/108 (96%)		
≥10^6^	112/118 (95%)		

**Table 4:** Adverse events frequency and severity.

	SOF+DCV N=96	SOF+LDV N=99	SOF+VEL N=31	Total N=226
Fatigue	18(19%)	34(34%)	6(19%)	58(26%)
Diarrhea	8(8%)	13(13%)	2(6%)	23(10%)
Headache	16(17%)	14(14%)	2(6%)	33(15%)
Nausea	10(10%)	6(7%)	3(10%)	19(8%)
Vomiting	4(4%)	7(7%)	2(6%)	13 (6%)
Insomnia	7(7%)	14(14%)	4(13%)	25(11%)
Dizziness	6(6%)	21(21%)	3(10%)	30(13%)
Cough	6(6%)	12(12%)	5(16%)	23(10%)
Adverse event leading to discontinuation	0	0	0	0
HCC occurrence during therapy	0	0	0	0
HCC occurrence during 12 weeks of follow-up period	0	0	0	0

## Data Availability

The data used to support the findings of this study are available from the corresponding author upon request.
